# Multilayer perceptron-based literature reading preferences predict anxiety and depression in university students

**DOI:** 10.3389/fpsyg.2024.1425471

**Published:** 2024-07-31

**Authors:** Yamei Liu

**Affiliations:** School of Languages and Cultures, Shanghai University of Political Science and Law, Shanghai, China

**Keywords:** literature reading preferences, anxiety, depression, university students, multilayer perceptron (MLP) neural network, mental health prediction

## Abstract

**Objective:**

This study aims to precisely model the nonlinear relationship between university students’ literature reading preferences (LRP) and their levels of anxiety and depression using a multilayer perceptron (MLP) to identify reading-related risk factors affecting anxiety and depression among university students.

**Methods:**

In this cross-sectional study, an internet-based questionnaire was conducted among 2,092 undergraduate students (aged 18–22, 62.7% female, from seven provinces in China). Participants completed a customized questionnaire on their LRP, followed by standardized assessments of anxiety and depression using the Generalized Anxiety Disorder 7-item Scale and the Beck Depression Inventory, respectively. An MLP with residual connections was employed to establish the nonlinear relationship between LRP and anxiety and depression.

**Results:**

The MLP model achieved an average accuracy of 86.8% for predicting non-anxious individuals and 81.4% for anxious individuals. In the case of depression, the model’s accuracy was 90.1% for non-depressed individuals and 84.1% for those with depression. SHAP value analysis identified “Tense/Suspenseful-Emotional Tone,” “War and Peace-Thematic Content,” and “Infrequent Reading-Reading Habits” as the top contributors to anxiety prediction accuracy. Similarly, “Sad-Emotional Tone Preference,” “Emotional Depictions-Thematic Content,” and “Thought-Provoking-Emotional Tone” were the primary contributors to depression prediction accuracy.

**Conclusion:**

The MLP accurately models the nonlinear relationship between LRP and mental health in university students, indicating the significance of specific reading preferences as risk factors. The study underscores the importance of literature emotional tone and themes in mental health. LRP should be integrated into psychological assessments to help prevent anxiety and depression among university students.

## 1 Introduction

Literature, integral to human culture, profoundly influences individuals and society (Balca, 2023). Reading preferences reveal cultural backgrounds and emotional experiences, offering data for psychological research (Moghaddam, 2004). Various literary types impact cognitive processes differently (Kalkbrenner et al., 2021), enhancing emotional empathy and social cognition (Hou et al., 2022). Deep reading enriches emotional understanding and emotional intelligence (Donaldson and Ko, 2010). Preferences for modernist literature correlate with openness and creativity (Teng, 2017), while skim reading links to pragmatic traits (Xiang et al., 2021). Personalized reading trends reflect cultural and emotional needs (Dani and Koenig, 2008), from complex narratives to straightforward plots.

Mental health issues among university students are increasingly serious, with rising rates of anxiety and depression (Auerbach, 2018). Factors contributing to this include competitive academic environments (Xu, 2022), job uncertainty (Song, 2021), and students’ adaptability challenges (Mao and Yang, 2024). Academic stress, interpersonal conflicts, and personal traits also influence negative emotions (Joseph et al., 1992; Zhong, 2022). While medication remains a primary treatment, it has limitations like side effects and dependency (Jauregui, 2002). Non-pharmacological approaches, particularly exercise, are effective in alleviating symptoms (Domínguez et al., 2015), yet research on these methods remains limited. Literature’s potential as a therapeutic tool also warrants further exploration in psychological studies.

In addition, Liu et al. (2024) longitudinal study also demonstrated the changes in anxiety and depression in the Chinese college student population, and further illustrated the relationship between negative emotions and self-perceptions of college students, and explored the long-term trends of depression and anxiety in the Chinese college student population through long-term tracking observations. The study can help understand the evolution of these emotional problems over time and may involve relationships with self-perception, coping strategies, and other factors. In addition, Liu et al. (2023) study found that gender, relationship with classmates, hometown location, BMI, and sleep duration were correlates that influenced different trajectories of depression development. In contrast, gender, extraversion, number of siblings, father’s education, and sleep duration were associated with different trajectories of anxiety development. In addition, hometown location, short and long sleep duration had significant effects on the categorization of different trajectories of stress development. These findings suggest that categorizing the developmental trajectories of depression, anxiety, and stress and identifying associated preventive measures is of practical relevance for targeted students. Early psychological intervention is especially important for students who may experience high or low mood shifts.

In the field of mental health, literature reading is regarded as an effective psychological adjustment tool. Different literature themes and reading styles have a significant impact on the mental health of university students. Svanda (2005) study found that reading positive and affirmative literature can enhance mood and self-identity, while over-indulgence in pessimistic or realistic works may exacerbate readers’ negative emotions (Creaven et al., 2023). Literature has the potential for emotional inspiration and mood regulation, but different types and styles of literature may have different effects. Some studies have noted that reading literature can help people better understand emotions and gain insights from them (Walker et al., 2023). However, some studies have also found that some literature may trigger negative emotions or emotional distress (Walker et al., 2023).

A multilayer perceptron (MLP) is an artificial neural network, which has been widely used in practical applications in a variety of fields such as image recognition, speech processing, and financial forecasting (Jiang et al., 2023; Wu, 2024). In recent years, its applications in health sciences have also shown significant growth, especially in disease diagnosis and biomarker discovery. However, there is still relatively little research at the intersection of sport science and mental health, particularly in the use of physical activity data to predict psychological states such as anxiety and depression. This suggests that there is significant potential for innovation in exploring and applying the use of MLP within this field.

In this study, we used a multilayer perceptron (MLP), a feed-forward artificial neural network model, to predict the mental health status of university students based on their literature reading preferences. The innovation of this approach is that it combines psychology, literature studies and artificial intelligence techniques in a first attempt to predict anxiety and depression in university students by analyzing literature reading preferences. Therefore, it is of great significance to conduct an in-depth study on the impact of literature reading on university students’ mental health. We selected different literary reading styles as predictors of influence factors and designed a questionnaire for literary reading preferences that passed the reliability and validity tests. This is because the Literary Reading Preferences Scale covers a number of dimensions, including literary genre preferences, thematic content preferences, reading context preferences, affective tone preferences, reading habits, material selection preferences, and reading purposes. These factors not only comprehensively reflect an individual’s preferences and choices of literature, but also allow for an in-depth exploration of reading habits and their potential impact on mental health. Previous studies have shown that literary reading is not only a leisure activity, but it is also closely related to an individual’s mental health. Different genres of literature and emotional responses may have different effects on mood and mental states. Therefore, an in-depth understanding of these preferences is important for predicting and understanding the occurrence of anxiety and depression in college students. In summary, the selection of these factor dimensions can effectively help reveal how literature reading preferences are associated with college students’ mental health problems and provide a scientific basis for developing targeted interventions. This comprehensive study is expected to provide new perspectives and strategies for the prevention and treatment of mental health problems among university students. Through this new avenue of research, we hope to effectively reduce the prevalence of anxiety and depression in the university student population, thereby enhancing their learning efficiency and quality of life.

## 2 Participants and methods

### 2.1 Participants

This study commenced on May 1, 2023, focusing primarily on undergraduate students aged 18 to 22. The questionnaires were designed and collected using the Wenjuanxing online platform, a web-based system for questionnaire administration, collection, and aggregation. The URL links and QR codes for the questionnaires were distributed via email and the WeChat app to university counselors, who then forwarded them to students for voluntary completion. Through this method, 2,092 valid questionnaires were collected from seven provinces (municipalities) in China, including Beijing, Tianjin, Shaanxi, Shanghai, Wuhan, Sichuan, and Liaoning. The sample consisted of students from four northern provinces (municipalities) and three southern provinces (municipalities). All participating students were enrolled in four-year comprehensive universities.

This study received approval from the Ethics Committee of Shanghai University of Political Science and Law (approval number: SH20240126). At the beginning of the questionnaire, all participants were informed about the potential risks and benefits of participating in the study. All participants provided signed informed consent before participating. All methods in this study were carried out in accordance with the relevant guidelines and regulations of the “Declaration of Helsinki”.

### 2.2 Screening and quality control of valid questionnaires

To eliminate questionnaires with potential technical errors, the study employed several exclusion criteria: (1) response time shorter than 30 s; (2) multiple submissions from the same IP address, with all submissions from that IP address being discarded; and (3) IP addresses not matching the province of the university.

Subsequently, the study excluded questionnaires from participants outside the 18–22 age range, those who reported reading infrequently or monthly, retaining only participants with daily or weekly reading habits. Additionally, individuals with severe mental health issues, such as schizophrenia, organic mental disorders, and those currently taking anti-anxiety and antidepressant medications, were excluded. Initially, 2,551 questionnaires were collected. After applying the exclusion criteria, 2,092 valid questionnaires remained, resulting in an 82% validity rate.

### 2.3 Study design

This study employed a cross-sectional design. All tools used in this study were self-report questionnaires. Initially, demographic information of the participants was collected, including gender, academic year, only-child status, and monthly family income. Additionally, an open-ended question asked participants whether they had any diagnosed psychological disorders and whether they were on medication.

Following this, participants were required to complete two questionnaires consecutively: a customized questionnaire on their literature reading habits and preferences, and a standardized assessment of anxiety and depression. This sequential data collection method ensured temporal and spatial separation of responses, thereby reducing potential response biases.

### 2.4 Depression and anxiety assessment

In this study, anxiety levels were evaluated using the Generalized Anxiety Disorder 7-item Scale (GAD-7), while depressive levels were assessed using the Beck Depression Inventory II (BDI-II).

In this study, anxiety levels were evaluated using the Generalized Anxiety Disorder 7-item Scale (GAD-7), while depressive levels were assessed using the Beck Depression Inventory II (BDI-II). The GAD-7 comprises seven items, with participants rating the frequency of each symptom over the past 2 weeks on a 4-point Likert scale, ranging from 0 (not at all) to 3 (nearly every day). The cumulative scores are categorized into the following anxiety severity levels: scores of 0–4 indicate the absence of anxiety, and scores of 5 and above suggest mild to severe anxiety. The Chinese version of the GAD-7 has demonstrated high internal consistency, exhibiting excellent internal consistency in this sample (Cronbach’s alpha = 0.92).

The BDI-II consists of 21 items, each rated on a 4-point Likert scale ranging from 0 to 3, with total scores ranging from 0 to 63. Higher scores indicate more severe depressive symptoms. The total scores are classified as follows in this study: scores of 0–13 typically indicate the absence of depression, while scores of 14 and above indicate mild to severe depression. The Chinese version of the BDI-II has shown high internal consistency, exhibiting excellent internal consistency in this sample (Cronbach’s alpha = 0.89).

### 2.5 Literature reading preference measurement

In this study, we developed a comprehensive Literature Reading Preference Scale to precisely measure individual preferences across various literature dimensions. Utilizing a 5-point Likert scale, where “1” indicates “strong dislike” or “strong disagreement,” and “5” signifies “strong liking” or “strong agreement,” the scale comprises 7 dimensions and 42 items.

The scale covers several dimensions:

Literature Genre Preferences: Including 8 distinct categories such as fiction, poetry, drama, prose essays, science fiction, mystery or thriller genres, historical or biographical works, and online literature like romance and fantasy.
Thematic Content Preferences: Assessed across 7 thematic areas, ranging from love to war and peace, growth and education, nature and the environment, societal and cultural issues, psychological analysis, and depiction of emotions.
Reading Context Preferences: Encompassing 5 different settings, comprising a quiet home, a library or reading room, public transport, parks or outdoor settings, and bedtime rituals.
Emotional Tone Preferences: Categorized into 7 emotional responses that literature may evoke, such as relaxation, excitement, sadness, thought-provoking content, tension or suspense, inspiration, or a lack of specific emotional direction.
Reading Habits: Explored through 6 reflective questions pertaining to the frequency of daily reading, regularity of weekly and monthly reading engagements, occurrence of sporadic reading, and duration of reading sessions, differentiated into those exceeding 1 hour and those falling short of 30 min.
Material Selection Preferences: Based on 4 common criteria influencing the choice of reading material, including recommendations from booklists, personal preferences, popularity of bestsellers, and random selection.
Purpose of Reading: Evaluated through 5 intrinsic motivations, encompassing leisure and entertainment, academic learning and research, emotional resonance, quest for inspiration, and facilitation of social interaction and discussion.

In this sample, the scale demonstrated good internal consistency (Cronbach’s alpha = 0.87). To ensure the scale’s test–retest reliability and validity, we recruited 50 participants who completed the assessment twice over a three-week interval. The test–retest reliability for each dimension ranged from 0.88 to 0.92. Confirmatory factor analysis indicated a good fit (CFI = 0.91), suggesting that the scale has strong structural validity.

### 2.6 Multilayer perceptron architecture

This study employed a multilayer perceptron (MLP) to explore the relationship between literature reading preferences and anxiety and depression among college students. Unlike traditional linear regression or logistic regression, MLPs can effectively capture non-linear relationships between variables, potentially achieving higher model accuracy. By examining the high-contribution variables identified by the model, we can effectively explore the literature reading preferences that influence anxiety and depression.

**Figure 1:**
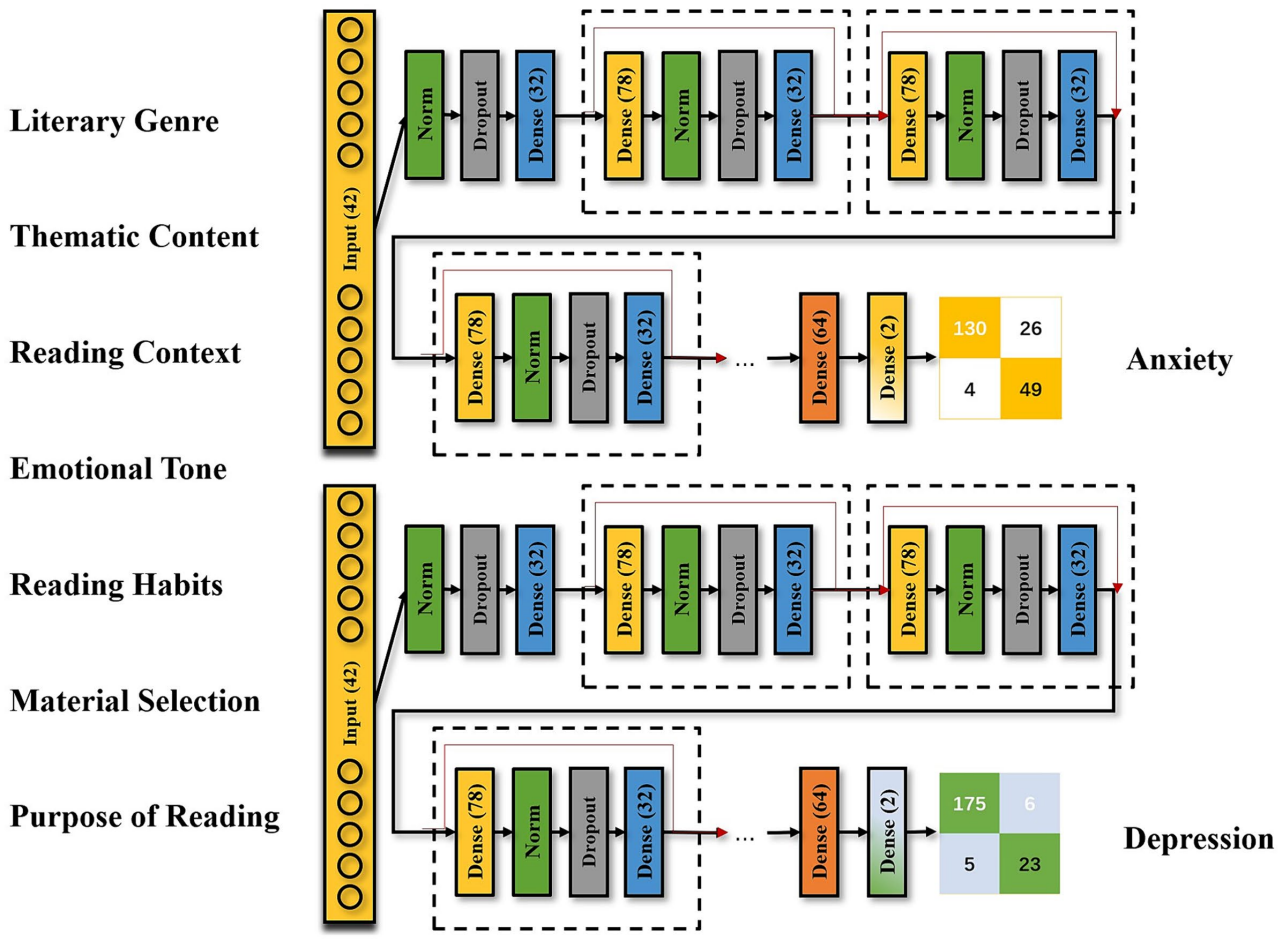
Multilayer perceptron architecture.

The MLP is well-suited for inputting multiple dimensions of features. Compared to random forests and support vector machines, the MLP model’s structure can be flexibly modified by adjusting the number of neurons in the hidden layers. The model comprises an input layer, multiple hidden layers, an output layer, and residual connections. The specific design is illustrated in Figure 1.

The input layer consists of 42 neurons, each receiving scores from the 42 items measuring literature reading preferences. The hidden layers are organized into modules, each containing four components: a dense layer, a normalization layer, a dropout layer, and another dense layer. Multiple modules are connected to a dense layer with 64 neurons, which is then connected to the output layer containing two neurons. All dense layers utilize the ReLU activation function. To address the vanishing and exploding gradient problems, residual connections were established between each module.

Initially, the number of modules, the number of neurons in the first and last dense layers of each module, and the dropout rate were undetermined and explored using the automated hyperparameter optimization tool Optuna. The final configuration included 20 modules, with 78 neurons in the first dense layer and 32 neurons in the last dense layer of each module, and a dropout rate of 0.55.

The model was trained using the Adam optimizer for 1,000 epochs with a batch size of 4. To enhance the interpretability of the model and explore the impact of variables on model predictions, SHAP values (SHapley Additive exPlanations) were introduced. SHAP values quantify the contribution of each individual feature to the model’s predictions, allowing us to rank and identify the significant literature reading variables that influence anxiety and depression.

### 2.7 Statistical analysis

Descriptive statistics were conducted for the entire sample. To evaluate the model, a ten-fold cross-validation method was employed. This process involved dividing the dataset into ten equal subsets, with each subset serving as the test set once while the remaining nine subsets constituted the training set. This iterative process ensured the robustness of the model.

For classification predictions, accuracy was used as the evaluation metric for model performance. Confusion matrices were generated for the results of each fold, and the model’s effectiveness was assessed by calculating the average accuracy from these confusion matrices.

To validate the performance of the neural network, support vector machines and random forests were also employed. A multifactor analysis of variance (ANOVA) was used to compare the accuracy differences among the various modeling methods, with post-hoc tests conducted using the Tukey test. The significance level for the two-tailed *p*-value was set at ≤0.05. Statistical analyses were performed using commercial software SPSS 25.

## 3 Results

### 3.1 Sample demographics

The sample included 780 males (37.3%) and 1,312 females (62.7%). The distribution across academic years was as follows: 862 freshmen (41.2%), 473 sophomores (22.6%), 571 juniors (27.3%), and 186 seniors (8.9%). Of these, 925 students (44.2%) resided in urban areas, and 1,167 students (55.8%) lived in rural areas. Anxiety was exhibited by 21.8% of the students, and 15.1% showed signs of depression.

### 3.2 Model performance

**Figure 2:**
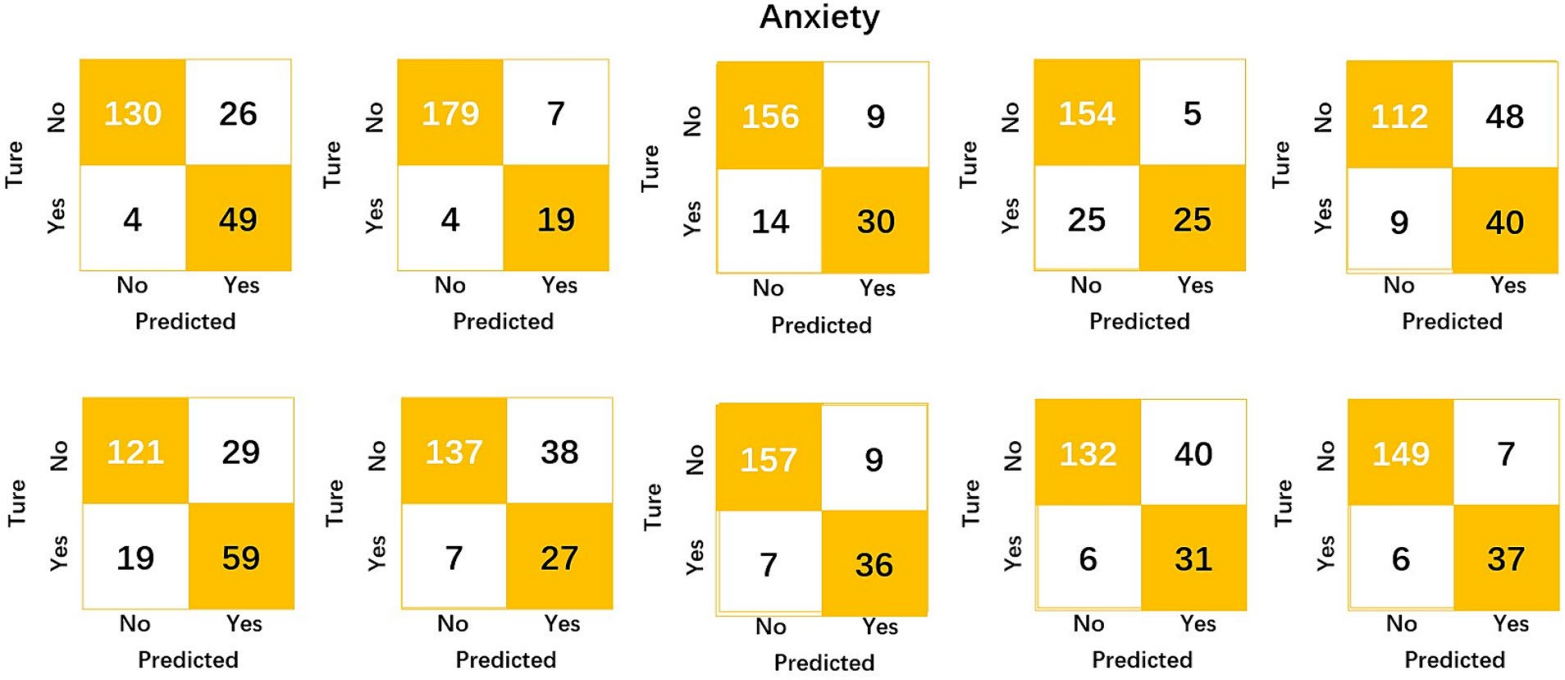
Confusion matrix for the prediction of anxiety by different folds.

The results of the studies in Table 1 and Figure 2 indicate that the average accuracy of MLP prediction of not anxious was 86.8% and the average accuracy of prediction of anxious was 81.4%.

**Table 1:** Predictive accuracy of different folds for anxiety.

	Anxiety	
Accuracy	No	Yes
Fold 1	83.3	92.4
Fold 2	96.2	82.6
Fold 3	94.5	68.1
Fold 4	96.8	81.6
Fold 5	70.0	81.6
Fold 6	80.6	75.6
Fold 7	82.0	79.4
Fold 8	94.5	83.7
Fold 9	76.7	83.7
Fold 10	95.5	86.0
Average	86.8	81.4

**Figure 3:**
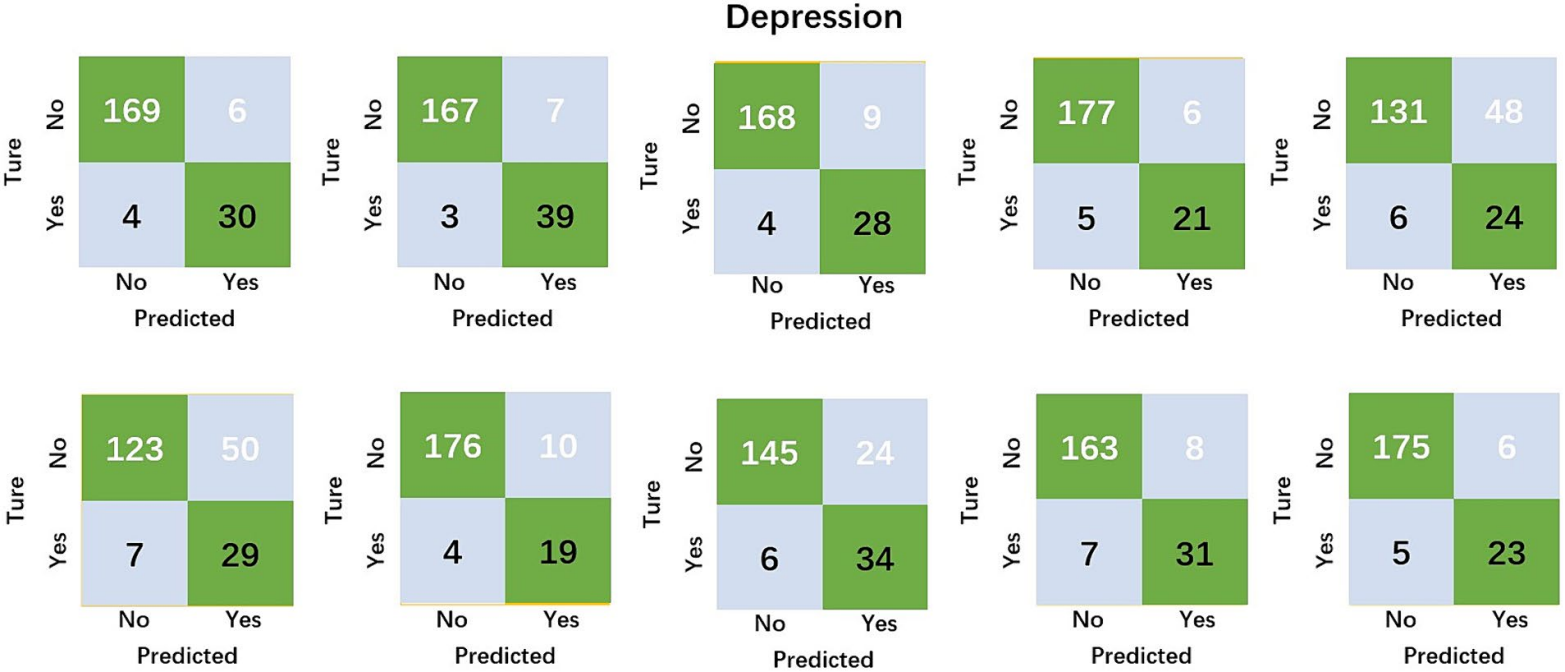
Confusion matrix for the prediction of depression by different folds.

The results of the study in Table 2 and Figure 3 indicate that MLP had an average accuracy of 90.1% for the prediction of non-depression and 84.1% for the prediction of depression.

**Table 2:** Predictive accuracy of different folds for depression.

	Depression	
Accuracy	No	Yes
Fold 1	96.5	88.2
Fold 2	95.8	92.8
Fold 3	94.9	87.5
Fold 4	96.7	80.7
Fold 5	73.2	80.0
Fold 6	71.1	80.5
Fold 7	94.6	82.6
Fold 8	85.7	85.0
Fold 9	95.3	81.5
Fold 10	96.6	82.1
Average	90.1	84.1

### 3.3 Analysis of high-contributing variables

**Figure 4:**
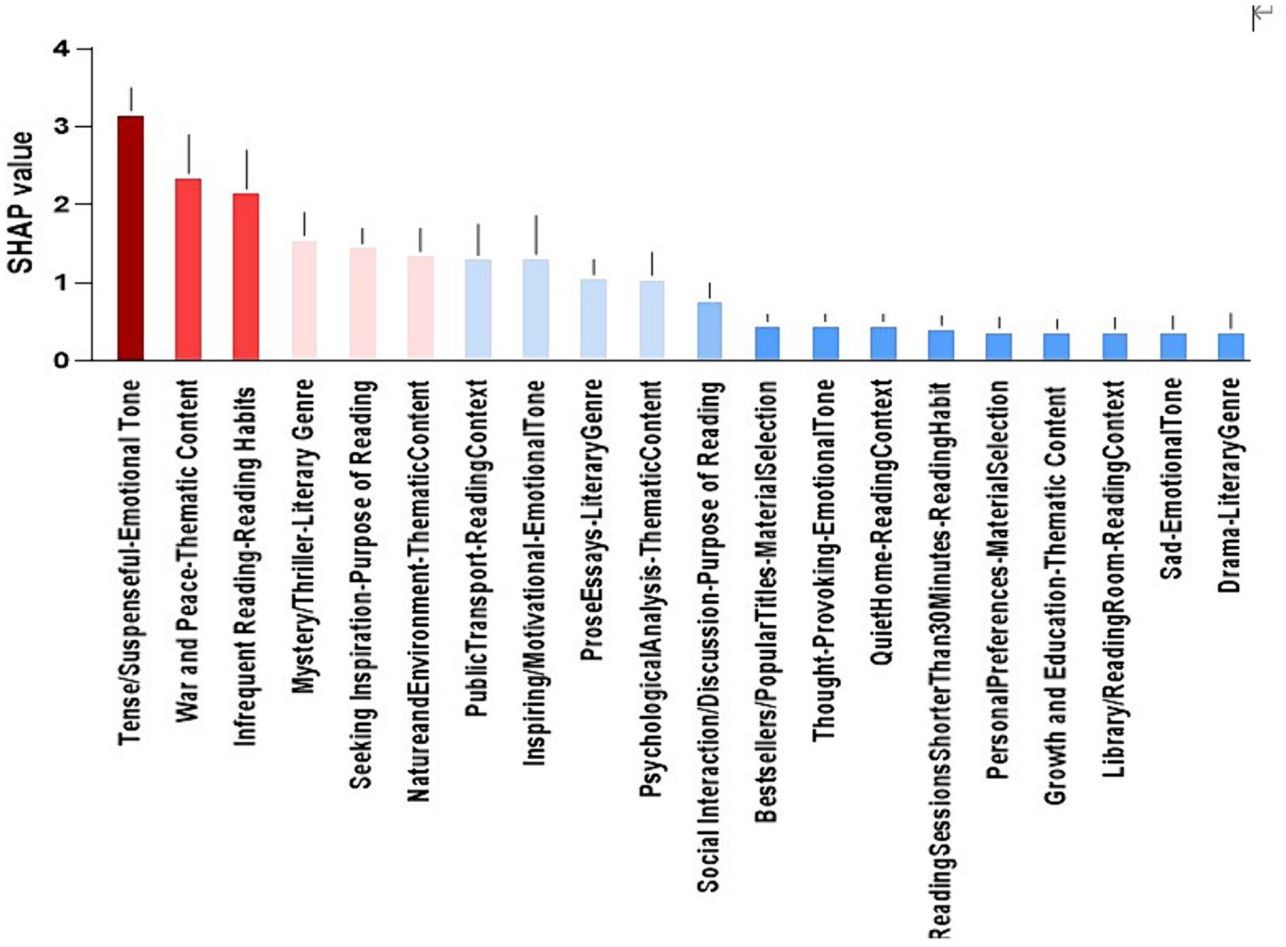
Ranking of anxiety prediction accuracy contributions.

The results of Figure 4 show that the Top 3 highest contributors to the prediction accuracy of anxiety were “Tense/Suspenseful-Emotional Tone,” “War and Peace-Thematic-Content” and “Infrequent Reading-Reading Habits.”

**Figure 5:**
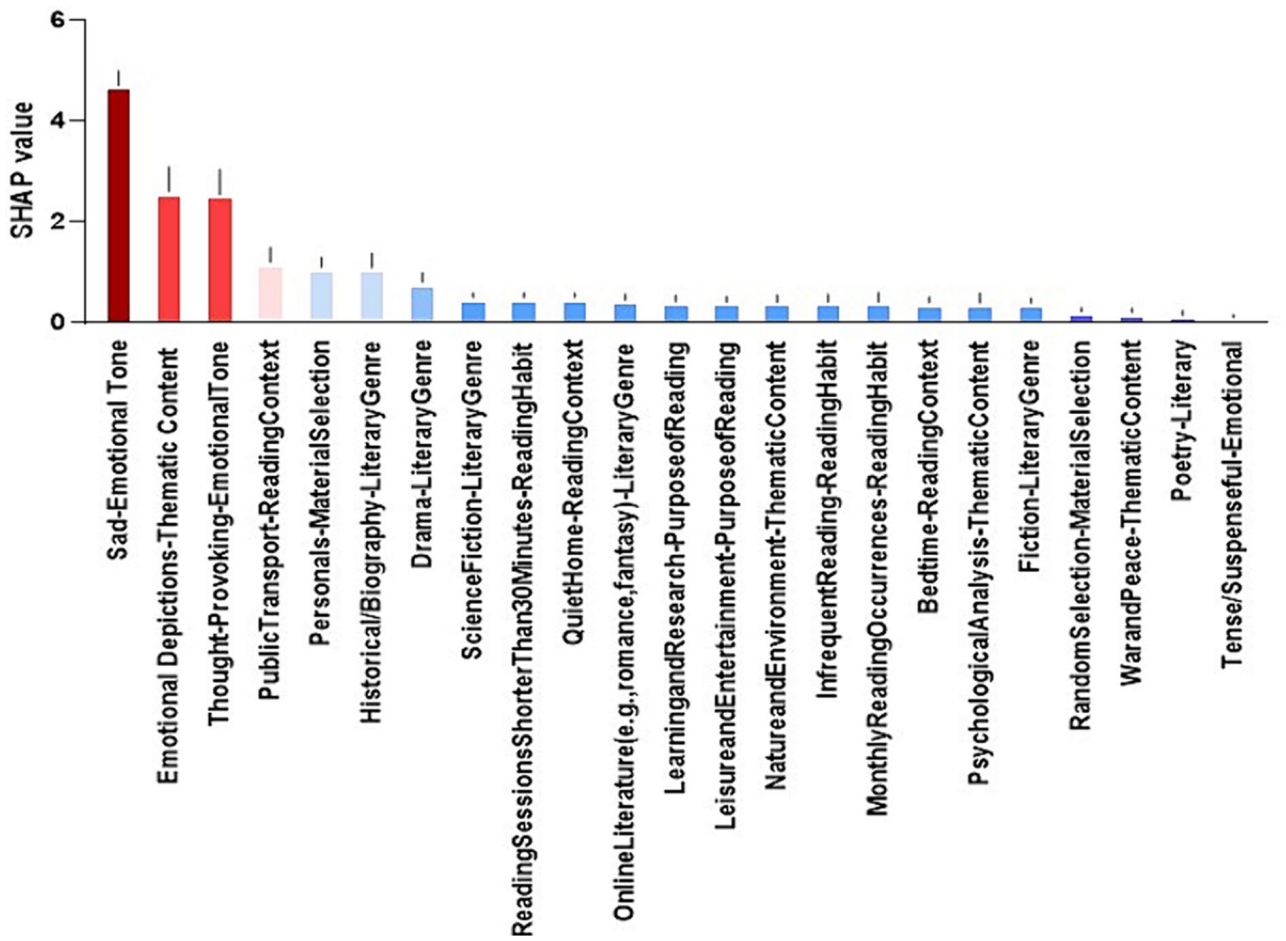
Ranking of depression prediction accuracy contributions.

The results in Figure 5 show that the Top 3 contributors to the prediction accuracy of depression are “Sad-Emotional Tone Preference,” “Emotional Depictions-Thematic-Content “and Thought Provoking-Emotional Tone.

### 3.4 Models comparison

Table 3 shows that the MLP significantly outperforms the SVM in predicting anxiety. In terms of predicting depression, the MLP also significantly outperforms both the SVM and the Random Forest models.

**Table 3:** Comparison of accuracy between MLP and other models.

	Anxiety	Depression
	Classifications accuracy	Classifications accuracy
SVM	63.7% ± 5.8%	61.4% ± 10.4%
Random forest	82.9% ± 2.6%^a^	72.3% ± 3.7%
MLP	84.1% ± 11.2%^a^	87.1% ± 11.3%^a^^b^
*F*	21.37	19.68
*p*	0.000	0.000

SVM represents support vector machine; MLP represents multilayer perceptron.

a Indicates that the difference is significant when compared to SVM.

b Indicates that the difference is significant when compared to random forest.

## 4 Discussion

The study included a total of 2,092 university students, consisting of 780 males (37.3%) and 1,312 females (62.7%). The distribution across academic years was as follows: 862 freshmen (41.2%), 473 sophomores (22.6%), 571 juniors (27.3%), and 186 seniors (8.9%). Geographically, 925 students (44.2%) resided in urban areas, while 1,167 students (55.8%) lived in rural areas. The prevalence of anxiety was observed in 21.8% of the students, and 15.1% exhibited signs of depression. These figures are consistent with other global and regional findings that emphasize the mental health challenges that college students may face amidst academic pressures, life adjustments, and social contextual changes.

In this study, the MLP model predicted that literature reading preferences affect anxiety and depression among university students found that the top three contributors to anxiety prediction accuracy were “Tense/Suspenseful-Emotional Tone,” “War and Peace-Thematic Content,” and “Infrequent Reading-Reading Habits.” These factors had a greater impact on the accuracy of anxiety prediction. This result suggests that emotional and thematic content in literature plays an important role in the prediction of anxiety state. Regarding the effect of reading styles on anxiety in university students, the characteristics of different reading styles and their impact on mental health can be analyzed in depth. This is in line with Arslan et al. (2022) study, which found that reading styles in the emotional suspense category may increase anxiety because they often engage readers by triggering a tense atmosphere or an unsolved mystery, which may make readers tense and uneasy during the reading process. On the contrary, some relaxing and enjoyable reading styles may reduce anxiety, such as humor-based works or heartwarming stories, which are more inclined to bring a pleasant and relaxing experience (Green, 2022). Further exploration of the effects of literary reading preferences on anxiety and depression highlights the complex interplay between emotional engagement and mental health outcomes. Findings are consistent with existing literature (Ikeda, 2023), highlighting that literature characterized by tension or suspense increases anxiety levels due to its ability to induce suspense and unease. Conversely, humorous or deeply moving narrative genres may promote relaxation and reduce anxiety. In addition, infrequent reading habits were found to be a significant predictor, suggesting that frequency of exposure to literature also plays a crucial role in mental health. This is in line with research emphasizing the therapeutic effects of a consistent reading habit on reducing stress and improving emotional well-being. This phenomenon may be related to the relationship between perceived stress and self-efficacy, which may cause a rise in perceived stress when reading content with a stressful/suspenseful-emotional tone, which in turn affects college students’ psychological mood, which is consistent with Liu’s study (Liu et al., 2024). He concluded that there is a significant correlation between perceived stress and self-efficacy among college students, which in turn affects mood changes. In addition, the reading habits of university students may also have an impact on their mental health. Infrequent reading may lead to insufficient knowledge base and incomplete information acquisition, which may increase anxiety, as a lack of coping ability in the face of various problems may trigger anxiety. Regular reading, on the other hand, can enhance an individual’s cognitive ability and emotional regulation, which can help reduce anxiety (Domschke and Ebert, 2024). Based on the findings of this paper, suggestions for university students to read may include choosing more relaxing and positive literature and avoiding overly tense suspenseful or depressing content; at the same time, encouraging the formation of good reading habits and maintaining the habit of regular reading in order to promote mental health. These recommendations also coincide with the results of past studies, such as who found that violent or suspenseful reading is more likely to trigger anxiety and tension than light and enjoyable reading. Therefore, the state of mental health of university students can be better maintained by choosing reading styles that are appropriate for individual mental health.

On the other hand, this study found that the prediction of literature reading preferences using the MLP model can effectively predict university students’ depression. The top three predictors of university students’ depression were found to be “Sad-Emotional Tone Preference,” “Emotional Depictions-Thematic Content,” and Thought-Provoking-Emotional Tone, respectively. The results of this study reveal the possibility of a close relationship between literature reading preferences and university students’ depression. The possibility of a strong link between literature reading preferences and depressed mood among university students. Specifically, sad-emotional, emotionally depicted, and thought-provoking literature had a significant impact on university students’ depressed mood. Expanding on this, literature has long been recognized for its potential to influence emotional states and mental health outcomes. According to previous research, narratives that evoke sadness or depict emotional struggles can resonate deeply with readers, potentially exacerbating feelings of sadness or melancholy. For instance, novels dealing with themes of loss or heartbreak might evoke empathetic responses that mirror the characters’ emotional turmoil, thereby intensifying negative emotions in susceptible individuals. Moreover, the thematic content of literature plays a pivotal role in shaping readers’ emotional responses. Stories that explore complex human emotions or existential dilemmas, such as those found in philosophical literature or psychologically introspective novels, may prompt deep reflection and introspection. While this engagement can be enriching for some readers, it may also evoke contemplation of existential concerns or personal challenges, potentially contributing to feelings of melancholy or depression (Moghaddam, 2004). The identification of thought-provoking literature as a predictor of depression highlights the importance of cognitive engagement in literary experiences. Works that challenge conventional beliefs or provoke intellectual curiosity can stimulate critical thinking and self-reflection, but they may also lead to rumination on unresolved issues or existential angst, which are common precursors to depressive symptoms (Hou et al., 2022).

另外, “Sad-Emotional Tone Preference,” a reading preference that may make university students more vulnerable to negative emotions. When they are immersed in literature with sad emotions, they may develop empathy and emotional resonance, which may deepen their own depressive mood. This is in line with Hevern (2022) study, who found that reading stories about loss, separation, or frustration may trigger negative emotions within university students, leading to more depressed moods. In addition, the reading content of “Emotional Depictions-Thematic Content” can deeply touch the readers’ hearts. However, for university students who are more emotionally sensitive, over-involvement in such works may lead to mood swings (Lim, 2022). If the Thematic Content involves negative emotions such as pain, struggle or helplessness, it may aggravate university students’ depression (Solity, 2022). This may be related to the fact that reading content influences personality, and different reading styles belong to the external environment, which affects the personality qualities of college students, which in turn affects their moods, in keeping with Liu et al. (2023) study, who argued that the external environment is an important factor affecting moods in college. In addition Thought-Provoking-Emotional Tone’s reading preference for the profundity and emotional tension of literature can often cause readers to think and empathize. However, this thought-provoking-emotional tone may cause university students to be overly contemplative and self-reflective, thus deepening their depressive mood. When university students overthink the dilemmas or challenges in their lives during the reading process, they may feel helpless and hopeless, which in turn exacerbates the development of depressive mood (Tao, 2022).

In addition, comparisons in this study showed that the MLP (Multilayer Perceptual Machine) model performed well in predicting anxiety and depression in college students. Specifically, the accuracy of MLP in anxiety prediction was 84.1% ± 11.2%, which was much higher than that of SVM (Support Vector Machine) at 63.7% ± 5.8% and Random Forest at 82.9% ± 2.6%. For depression prediction, the accuracy of MLP was 87.1% ± 11.3%, which was also significantly better than SVM’s 61.4% ± 10.4% and Random Forest’s 72.3% ± 3.7%. These results are not just statistical advantages, but also reflect the actual effectiveness of MLP when dealing with mental health prediction tasks. MLP, as a neural network-based model, is able to effectively utilize complex data patterns and nonlinear relationships between features, thus improving the accuracy and reliability of predictions. In contrast, SVM and Random Forest, although they perform well in some cases, have relatively limited ability to handle complex data relationships and high-dimensional nonlinear relationships between features. In addition, the study also demonstrated the significant advantage of MLPs in the prediction task through the statistical analysis of *F*-values and *p*-values, which showed statistically significant differences in accuracy of MLPs relative to other models. These findings not only emphasize the potential of the MLP as an efficient prediction tool, but also provide important empirical support for future applications in mental health support and intervention for college students.

In summary, literature reading preferences have a significant impact on anxiety and depression among university students. Through the prediction results of the MLP model, we found that specific types of emotional and thematic content play a key role in predicting anxiety and depression. Therefore, it is crucial for university students to choose appropriate reading materials and develop good reading habits. By choosing relaxing and positive works and maintaining a regular reading habit, university students can better maintain their mental health (Locher, 2022). In addition, reading content should also be targeted for individual emotional preferences to avoid overindulgence in works that trigger negative emotions. This study provides an important reference for the mental health of university students, and it is hoped that it can draw more attention and support from all sectors of society to the mental health of university students. The innovation of this study is that it combines literature reading preferences with neural network modeling, which is the first attempt to analyze literature reading preferences to predict university students’ anxiety and depression. The innovation and practicality of this research method provides new perspectives and strategies for the prevention and treatment of mental health problems. Through in-depth research on the impact of different types of literature on mental health and how individual literature reading preferences affect psychological states, more personalized and effective mental health interventions can be developed.

### 4.1 Strengths

1. This study is the first to combine literature reading preference with neural network modeling to predict anxiety and depression among university students by analyzing literature reading preference, which provides new perspectives and strategies for the prevention and treatment of mental health problems.
2. Through in-depth research on the effects of different types of literature on mental health and the effects of individual literature reading preferences on psychological states, more personalized and effective mental health interventions can be developed to provide better psychological support for university students. It also provides proper guidance for university students’ reading styles.

### 4.2 Limitations

This study only focused on the relationship between literature reading preference and anxiety and depression and did not consider other possible influencing factors such as social support and personal experiences. Therefore, further research is needed to explore the impact of these factors on mental health.

## 5 Conclusion

The findings of this study suggest that anxiety and depression in university students can be predicted by analyzing literature reading preferences, providing them with the right reading style and psychological support. We recommend that university students choose more light-hearted and positive literature, avoid overly stressful and dull content, and encourage the formation of good reading habits. This innovative approach provides new research avenues and strategies in the field of mental health. By thoroughly investigating the effects of different types of literature on mental health and the impact of individual literature reading preferences on psychological states, we can develop more personalized and effective mental health interventions.

In addition, given the current widespread application of AI in the field of mental health, it is recommended that AI-assisted tools be incorporated into college students’ mental health intervention programs in order to provide more personalized, real-time psychological support and consulting services. Combining AI technology and big data analysis, an intelligent mental health monitoring system can be developed to monitor changes in the psychological status of college students in real time, detect potential mental health problems in a timely manner and provide appropriate interventions. We found a close relationship between reading preference and college students’ anxiety and depression through AI, and suggest that future research focuses on the impact of different reading styles on mental health in order to develop more effective psychological interventions.

## Data availability statement

The original contributions presented in the study are included in the article/supplementary material, further inquiries can be directed to the corresponding author.

## Ethics statement

The studies involving humans were reviewed and approved by the Ethics Committee of the China National Rehabilitation Center (No. S20240206). The studies were conducted in accordance with the local legislation and institutional requirements. The participants provided their written informed consent to participate in this study.

## Author contributions

YL: Writing – review & editing, Writing – original draft.
